# Personal

**Published:** 1907-01

**Authors:** 


### PERSONAL.

LIEUTENANT-COLONEL EUGENE A. SMITH, surgeon Fourth Brigade N. Y. N. G., of Buffalo, has been appointed a member of the examining board for medical officers in the National Guard organisation. No more fitting appointment could be made. Dr. Smith is a surgeon of distinction, a veteran of the Spanish war, and a citizen of prominence. He is the author of the chapter on traumatic fevers in Keen's new surgery, the first volume of which is reviewed elsewhere in this issue.

---

DR. N. P. CARD of Utica, who was lately appointed assistant surgeon in the hospital of the State Soldiers' and Sailors' Home at Bath, has presented his resignation to Commandant Ewell, after having been on duty five days. He will accept a position in the Manhattan State Hospital. Dr. Carlton Foster, late of the state hospital at Cenral Islip, has been selected from the state civil service list to succeed Dr. Card.

---

Dr. Earl G. Danser, Medical Examiner for Erie County (in cases of sudden death) who has been ill for some time with blood poisoning contracted at an autopsy, having recovered, has returned to his official duties and resumed his professional practice.

---

Dr. Brooks H. Wells, editor of the American Journal of Obstetrics, has been elected president of the New York Obstetrical Society for the current year. The society has chosen wisely, Dr. Wells being one of its most prominent and capable members.

---

Dr. Irving W. Potter, of Buffalo, announces the removal of his office and residence from 806 Fillmore Avenue to 420 Franklin street, corner Virginia. Hours: 2-3 and 6-8 P. M. Telephones: Bell, Tupper 511; Frontier, 511.

---

Professor di Venezia, according to an announcement from Rome, probably will succeed the late Dr. Lapponi as titular pontifical physician. The salary paid to Dr. Laponi was $1,200.

---

Assistant Surgeon C. S. Ely, United States Navy, has been assigned to duty, under recent orders of the department, at the naval recruiting station in Buffalo.

---

Dr. John D'Arcy O'Brien, University of Buffalo, 1904, now of Lockport, has been appointed physician to the Niagara County jail.

